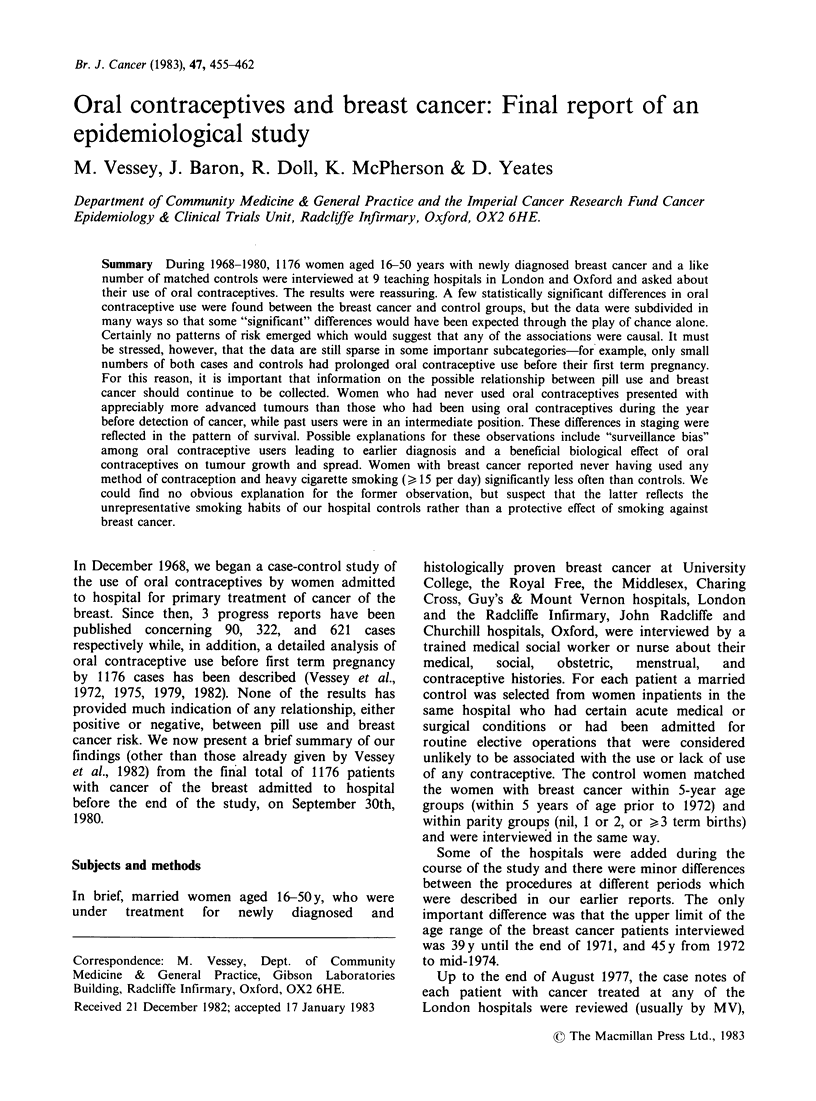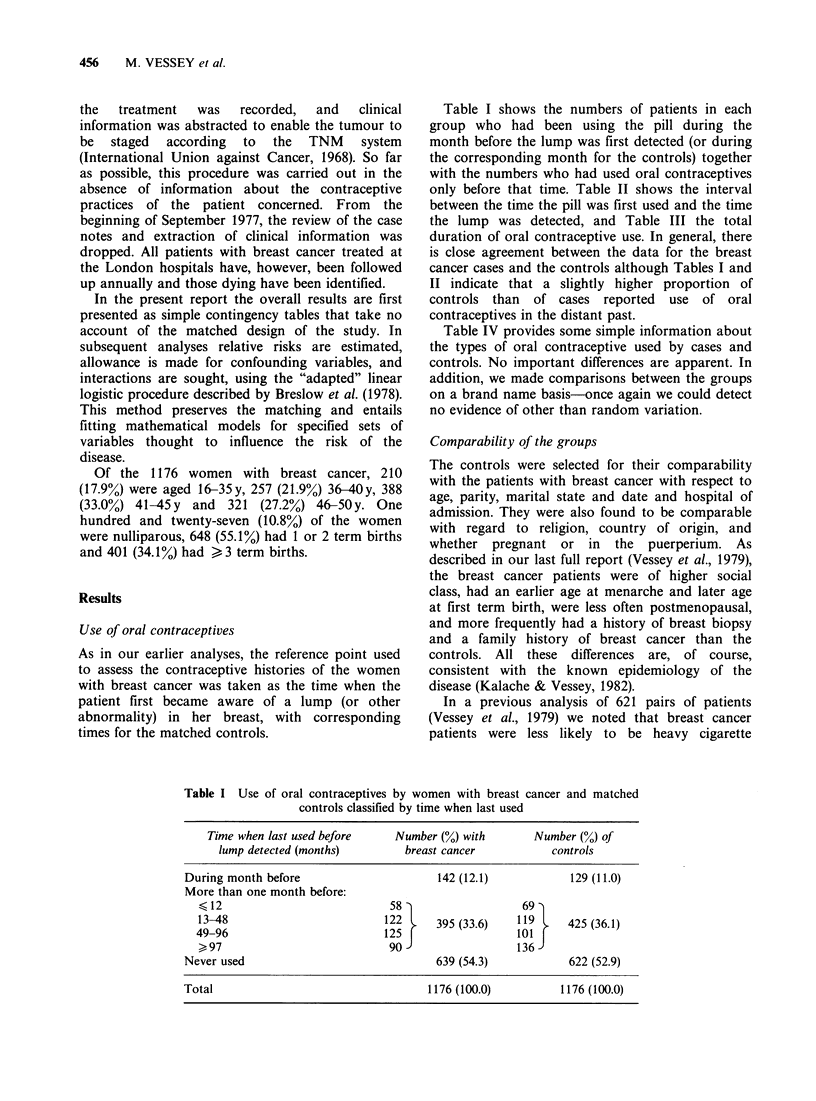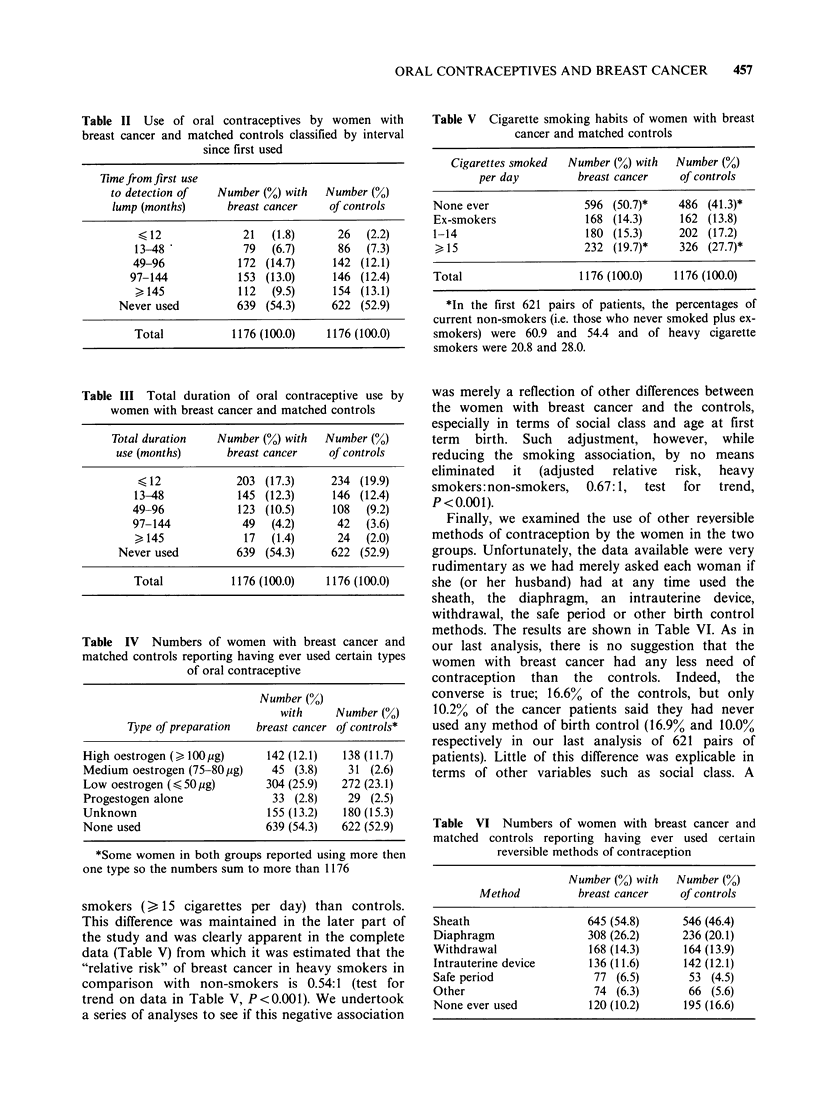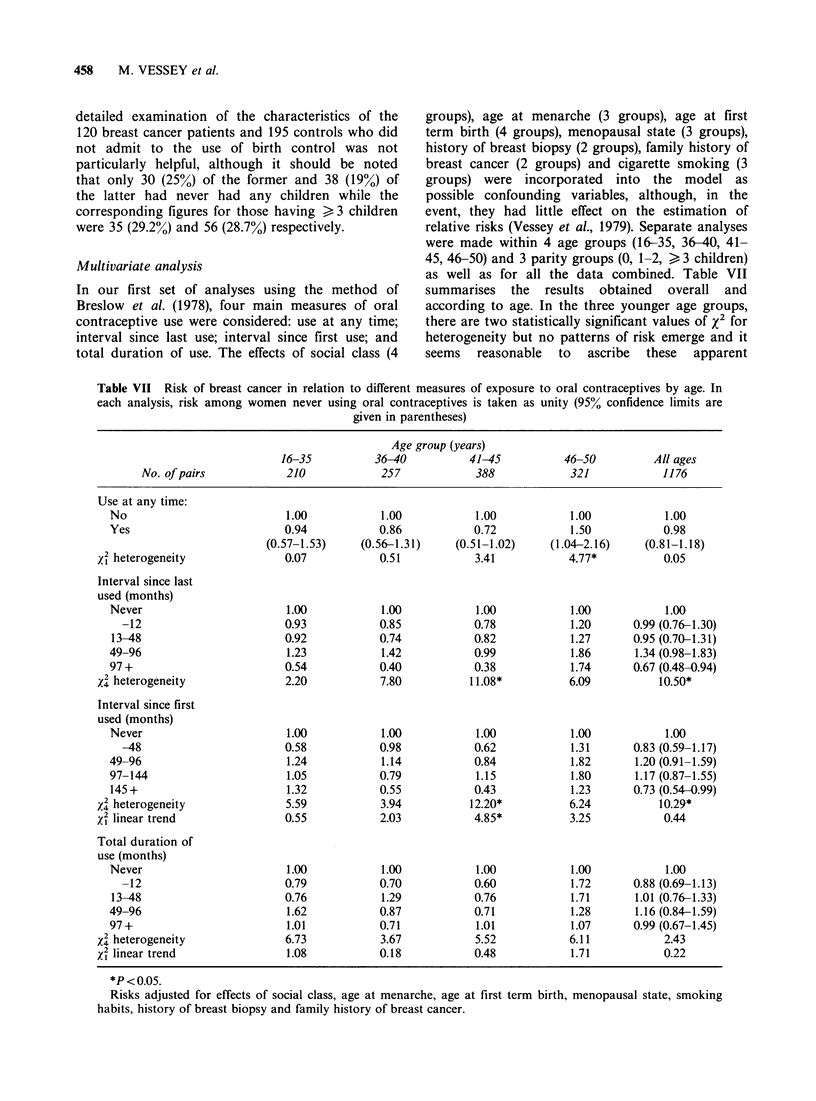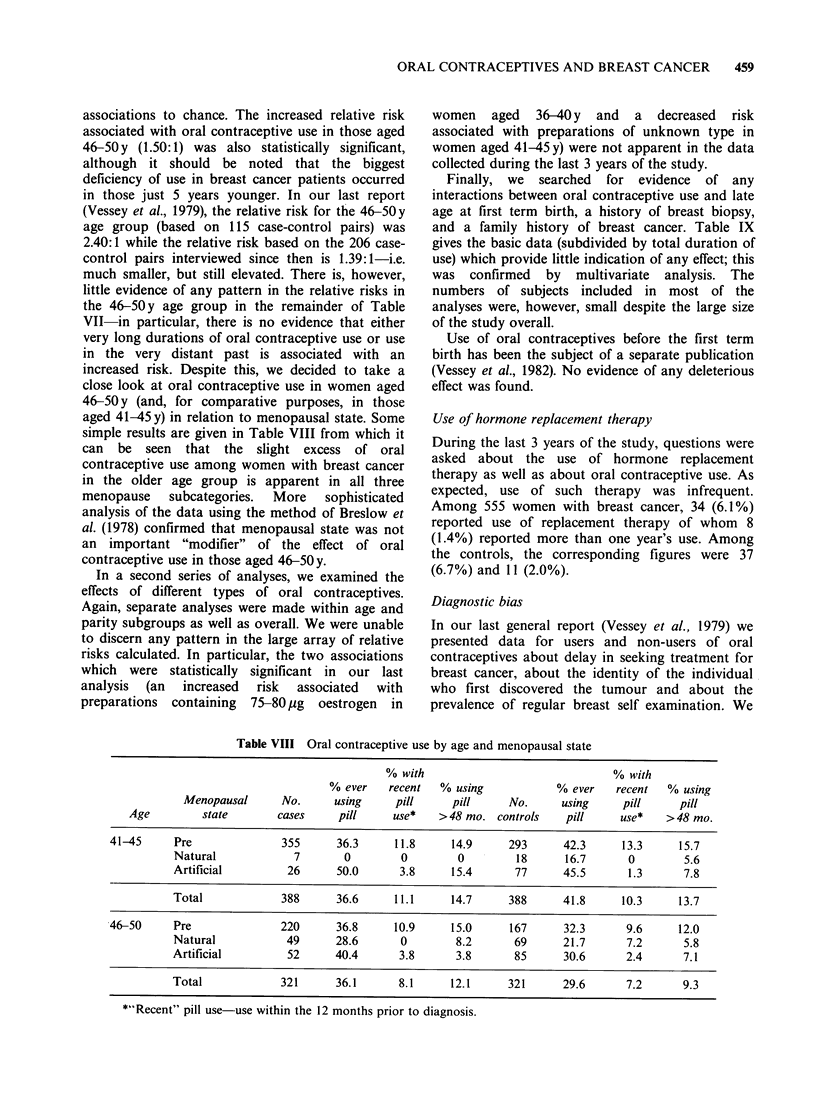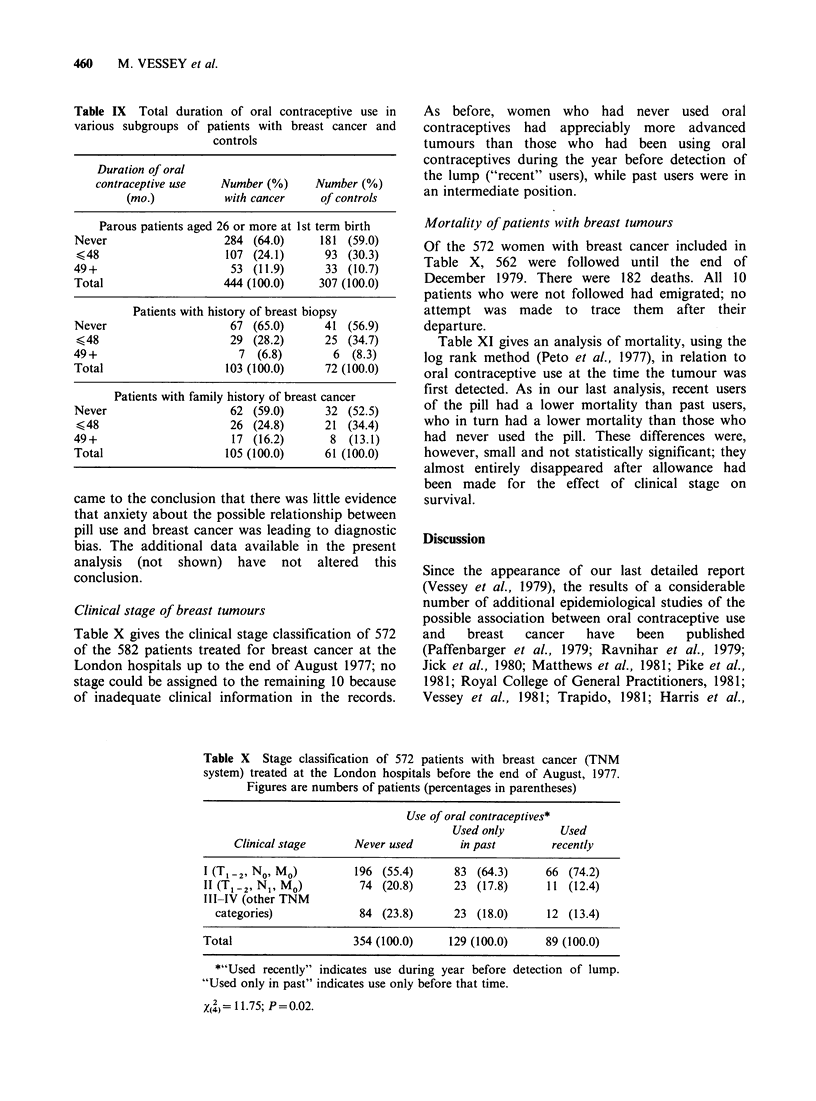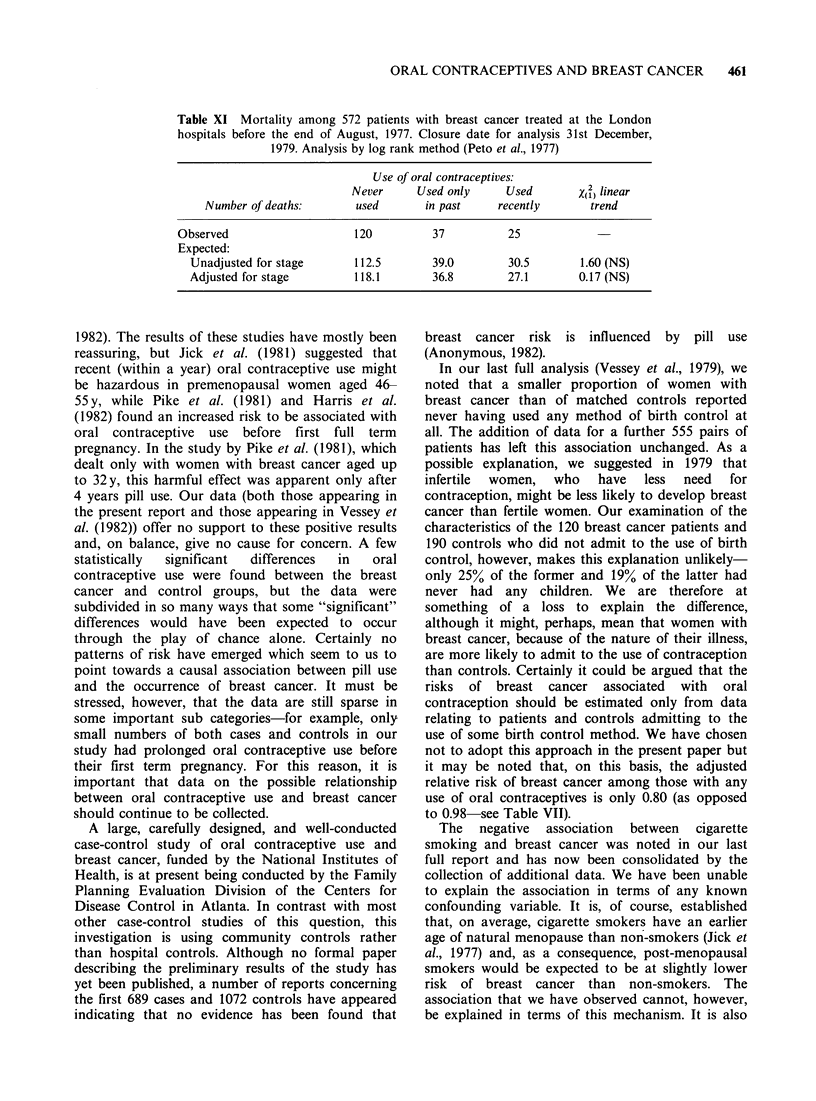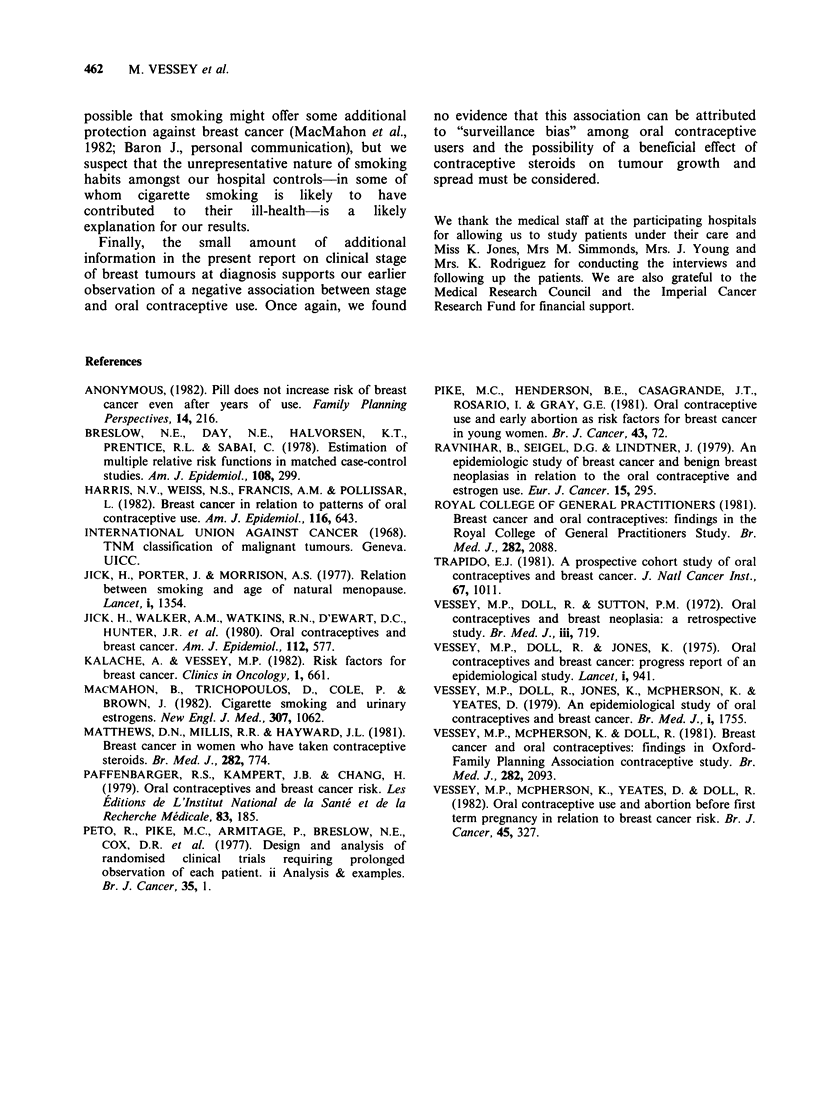# Oral contraceptives and breast cancer: final report of an epidemiological study.

**DOI:** 10.1038/bjc.1983.74

**Published:** 1983-04

**Authors:** M. Vessey, J. Baron, R. Doll, K. McPherson, D. Yeates

## Abstract

During 1968-1980, 1176 women aged 16-50 years with newly diagnosed breast cancer and a like number of matched controls were interviewed at 9 teaching hospitals in London and Oxford and asked about their use of oral contraceptives. The results were reassuring. A few statistically significant differences in oral contraceptive use were found between the breast cancer and control groups, but the data were subdivided in many ways so that some "significant" differences would have been expected through the play of chance alone. Certainly no patterns of risk emerged which would suggest that any of the associations were causal. It must be stressed, however, that the data are still sparse in some important subcategories--for example, only small numbers of both cases and controls had prolonged oral contraceptive use before their first term pregnancy. For this reason, it is important that information on the possible relationship between pill use and breast cancer should continue to be collected. Women who had never used oral contraceptives presented with appreciably more advanced tumours than those who had been using oral contraceptives during the year before detection of cancer, while past users were in an intermediate position. These differences in staging were reflected in the pattern of survival. Possible explanations for these observations include "surveillance bias" among oral contraceptive users leading to earlier diagnosis and a beneficial biological effect of oral contraceptives on tumour growth and spread. Women with breast cancer reported never having used any method of contraception and heavy cigarette smoking (greater than or equal to 15 per day) significantly less often than controls. We could find no obvious explanation for the former observation, but suspect that the latter reflects the unrepresentative smoking habits of our hospital controls rather than a protective effect of smoking against breast cancer.


*Br. J. Cancer* (1983), **47**, 455–462

### Oral contraceptives and breast cancer: Final report of an epidemiological study

M. Vessey, J. Baron, R. Doll, K. McPherson & D. Yeates

*Department of Community Medicine & General Practice and the Imperial Cancer Research Fund Cancer Epidemiology & Clinical Trials Unit, Radcliffe Infirmary, Oxford, OX2 6HE.*

**Summary** During 1968–1980, 1176 women aged 16–50 years with newly diagnosed breast cancer and a like number of matched controls were interviewed at 9 teaching hospitals in London and Oxford and asked about their use of oral contraceptives. The results were reassuring. A few statistically significant differences in oral contraceptive use were found between the breast cancer and control groups, but the data were subdivided in many ways so that some "significant" differences would have been expected through the play of chance alone. Certainly no patterns of risk emerged which would suggest that any of the associations were causal. It must be stressed, however, that the data are still sparse in some importanr subcategories—for example, only small numbers of both cases and controls had prolonged oral contraceptive use before their first term pregnancy. For this reason, it is important that information on the possible relationship between pill use and breast cancer should continue to be collected. Women who had never used oral contraceptives presented with appreciably more advanced tumours than those who had been using oral contraceptives during the year before detection of cancer, while past users were in an intermediate position. These differences in staging were reflected in the pattern of survival. Possible explanations for these observations include "surveillance bias" among oral contraceptive users leading to earlier diagnosis and a beneficial biological effect of oral contraceptives on tumour growth and spread. Women with breast cancer reported never having used any method of contraception and heavy cigarette smoking (⩾15 per day) significantly less often than controls. We could find no obvious explanation for the former observation, but suspect that the latter reflects the unrepresentative smoking habits of our hospital controls rather than a protective effect of smoking against breast cancer.

In December 1968, we began a case-control study of the use of oral contraceptives by women admitted to hospital for primary treatment of cancer of the breast. Since then, 3 progress reports have been published concerning 90, 322, and 621 cases respectively while, in addition, a detailed analysis of oral contraceptive use before first term pregnancy by 1176 cases has been described (Vessey *et al.*, 1972, 1975, 1979, 1982). None of the results has provided much indication of any relationship, either positive or negative, between pill use and breast cancer risk. We now present a brief summary of our findings (other than those already given by Vessey *et al.*, 1982) from the final total of 1176 patients with cancer of the breast admitted to hospital before the end of the study, on September 30th, 1980.

### Subjects and methods

In brief, married women aged 16–50 y, who were under treatment for newly diagnosed and histologically proven breast cancer at University College, the Royal Free, the Middlesex, Charing Cross, Guy's & Mount Vernon hospitals, London and the Radcliffe Infirmary, John Radcliffe and Churchill hospitals, Oxford, were interviewed by a trained medical social worker or nurse about their medical, social, obstetric, menstrual, and contraceptive histories. For each patient a married control was selected from women inpatients in the same hospital who had certain acute medical or surgical conditions or had been admitted for routine elective operations that were considered unlikely to be associated with the use or lack of use of any contraceptive. The control women matched the women with breast cancer within 5-year age groups (within 5 years of age prior to 1972) and within parity groups (nil, 1 or 2, or ⩾3 term births) and were interviewed in the same way.

Some of the hospitals were added during the course of the study and there were minor differences between the procedures at different periods which were described in our earlier reports. The only important difference was that the upper limit of the age range of the breast cancer patients interviewed was 39 y until the end of 1971, and 45 y from 1972 to mid-1974.

Up to the end of August 1977, the case notes of each patient with cancer treated at any of the London hospitals were reviewed (usually by MV),

Correspondence: M. Vessey, Dept. of Community Medicine & General Practice, Gibson Laboratories Building, Radcliffe Infirmary, Oxford, OX2 6HE.

Received 21 December 1982; accepted 17 January 1983

© The Macmillan Press Ltd., 1983

the treatment was recorded, and clinical information was abstracted to enable the tumour to be staged according to the TNM system (International Union against Cancer, 1968). So far as possible, this procedure was carried out in the absence of information about the contraceptive practices of the patient concerned. From the beginning of September 1977, the review of the case notes and extraction of clinical information was dropped. All patients with breast cancer treated at the London hospitals have, however, been followed up annually and those dying have been identified.

In the present report the overall results are first presented as simple contingency tables that take no account of the matched design of the study. In subsequent analyses relative risks are estimated, allowance is made for confounding variables, and interactions are sought, using the "adapted" linear logistic procedure described by Breslow et al. (1978). This method preserves the matching and entails fitting mathematical models for specified sets of variables thought to influence the risk of the disease.

Of the 1176 women with breast cancer, 210 (17.9%) were aged 16–35 y, 257 (21.9%) 36–40 y, 388 (33.0%) 41–45 y and 321 (27.2%) 46–50 y. One hundred and twenty-seven (10.8%) of the women were nulliparous, 648 (55.1%) had 1 or 2 term births and 401 (34.1%) had ⩾3 term births.

### Results

### *Use of oral contraceptives*

As in our earlier analyses, the reference point used to assess the contraceptive histories of the women with breast cancer was taken as the time when the patient first became aware of a lump (or other abnormality) in her breast, with corresponding times for the matched controls.

Table I shows the numbers of patients in each group who had been using the pill during the month before the lump was first detected (or during the corresponding month for the controls) together with the numbers who had used oral contraceptives only before that time. Table II shows the interval between the time the pill was first used and the time the lump was detected, and Table III the total duration of oral contraceptive use. In general, there is close agreement between the data for the breast cancer cases and the controls although Tables I and II indicate that a slightly higher proportion of controls than of cases reported use of oral contraceptives in the distant past.

Table IV provides some simple information about the types of oral contraceptive used by cases and controls. No important differences are apparent. In addition, we made comparisons between the groups on a brand name basis—once again we could detect no evidence of other than random variation.

### *Comparability of the groups*

The controls were selected for their comparability with the patients with breast cancer with respect to age, parity, marital state and date and hospital of admission. They were also found to be comparable with regard to religion, country of origin, and whether pregnant or in the puerperium. As described in our last full report (Vessey et al., 1979), the breast cancer patients were of higher social class, had an earlier age at menarche and later age at first term birth, were less often postmenopausal, and more frequently had a history of breast biopsy and a family history of breast cancer than the controls. All these differences are, of course, consistent with the known epidemiology of the disease (Kalache & Vessey, 1982).

In a previous analysis of 621 pairs of patients (Vessey et al., 1979) we noted that breast cancer patients were less likely to be heavy cigarette

**Table I** Use of oral contraceptives by women with breast cancer and matched controls classified by time when last used

| *Time when last used before lump detected (months)* | | *Number (%) with breast cancer* | | *Number (%) of controls* |
|---|---|---|---|---|
| During month before | | 142 (12.1) | | 129 (11.0) |
| More than one month before: | | | | |
| ⩽12 | 58 | 395 (33.6) | 69 | 425 (36.1) |
| 13–48 | 122 | | 119 | |
| 49–96 | 125 | | 101 | |
| ⩾97 | 90 | | 136 | |
| Never used | | 639 (54.3) | | 622 (52.9) |
| Total | | 1176 (100.0) | | 1176 (100.0) |

**Table II** Use of oral contraceptives by women with breast cancer and matched controls classified by interval since first used

| *Time from first use to detection of lump (months)* | *Number (%) with breast cancer* | *Number (%) of controls* |
|---|---|---|
| ⩽12 | 21 (1.8) | 26 (2.2) |
| 13–48 | 79 (6.7) | 86 (7.3) |
| 49–96 | 172 (14.7) | 142 (12.1) |
| 97–144 | 153 (13.0) | 146 (12.4) |
| ⩾145 | 112 (9.5) | 154 (13.1) |
| Never used | 639 (54.3) | 622 (52.9) |
| Total | 1176 (100.0) | 1176 (100.0) |

**Table III** Total duration of oral contraceptive use by women with breast cancer and matched controls

| *Total duration use (months)* | *Number (%) with breast cancer* | *Number (%) of controls* |
|---|---|---|
| ⩽12 | 203 (17.3) | 234 (19.9) |
| 13–48 | 145 (12.3) | 146 (12.4) |
| 49–96 | 123 (10.5) | 108 (9.2) |
| 97–144 | 49 (4.2) | 42 (3.6) |
| ⩾145 | 17 (1.4) | 24 (2.0) |
| Never used | 639 (54.3) | 622 (52.9) |
| Total | 1176 (100.0) | 1176 (100.0) |

**Table IV** Numbers of women with breast cancer and matched controls reporting having ever used certain types of oral contraceptive

| *Type of preparation* | *Number (%) with breast cancer* | *Number (%) of controls** |
|---|---|---|
| High oestrogen (⩾100 μg) | 142 (12.1) | 138 (11.7) |
| Medium oestrogen (75–80 μg) | 45 (3.8) | 31 (2.6) |
| Low oestrogen (⩽50 μg) | 304 (25.9) | 272 (23.1) |
| Progestogen alone | 33 (2.8) | 29 (2.5) |
| Unknown | 155 (13.2) | 180 (15.3) |
| None used | 639 (54.3) | 622 (52.9) |

*Some women in both groups reported using more then one type so the numbers sum to more than 1176

smokers (⩾15 cigarettes per day) than controls. This difference was maintained in the later part of the study and was clearly apparent in the complete data (Table V) from which it was estimated that the "relative risk" of breast cancer in heavy smokers in comparison with non-smokers is 0.54:1 (test for trend on data in Table V, $P<0.001$). We undertook a series of analyses to see if this negative association

**Table V** Cigarette smoking habits of women with breast cancer and matched controls

| *Cigarettes smoked per day* | *Number (%) with breast cancer* | *Number (%) of controls* |
|---|---|---|
| None ever | 596 (50.7)* | 486 (41.3)* |
| Ex-smokers | 168 (14.3) | 162 (13.8) |
| 1–14 | 180 (15.3) | 202 (17.2) |
| ⩾15 | 232 (19.7)* | 326 (27.7)* |
| Total | 1176 (100.0) | 1176 (100.0) |

*In the first 621 pairs of patients, the percentages of current non-smokers (i.e. those who never smoked plus ex-smokers) were 60.9 and 54.4 and of heavy cigarette smokers were 20.8 and 28.0.

was merely a reflection of other differences between the women with breast cancer and the controls, especially in terms of social class and age at first term birth. Such adjustment, however, while reducing the smoking association, by no means eliminated it (adjusted relative risk, heavy smokers:non-smokers, 0.67:1, test for trend, $P<0.001$).

Finally, we examined the use of other reversible methods of contraception by the women in the two groups. Unfortunately, the data available were very rudimentary as we had merely asked each woman if she (or her husband) had at any time used the sheath, the diaphragm, an intrauterine device, withdrawal, the safe period or other birth control methods. The results are shown in Table VI. As in our last analysis, there is no suggestion that the women with breast cancer had any less need of contraception than the controls. Indeed, the converse is true; 16.6% of the controls, but only 10.2% of the cancer patients said they had never used any method of birth control (16.9% and 10.0% respectively in our last analysis of 621 pairs of patients). Little of this difference was explicable in terms of other variables such as social class. A

**Table VI** Numbers of women with breast cancer and matched controls reporting having ever used certain reversible methods of contraception

| *Method* | *Number (%) with breast cancer* | *Number (%) of controls* |
|---|---|---|
| Sheath | 645 (54.8) | 546 (46.4) |
| Diaphragm | 308 (26.2) | 236 (20.1) |
| Withdrawal | 168 (14.3) | 164 (13.9) |
| Intrauterine device | 136 (11.6) | 142 (12.1) |
| Safe period | 77 (6.5) | 53 (4.5) |
| Other | 74 (6.3) | 66 (5.6) |
| None ever used | 120 (10.2) | 195 (16.6) |

detailed examination of the characteristics of the 120 breast cancer patients and 195 controls who did not admit to the use of birth control was not particularly helpful, although it should be noted that only 30 (25%) of the former and 38 (19%) of the latter had never had any children while the corresponding figures for those having ⩾3 children were 35 (29.2%) and 56 (28.7%) respectively.

*Multivariate analysis*

In our first set of analyses using the method of Breslow *et al.* (1978), four main measures of oral contraceptive use were considered: use at any time; interval since last use; interval since first use; and total duration of use. The effects of social class (4 groups), age at menarche (3 groups), age at first term birth (4 groups), menopausal state (3 groups), history of breast biopsy (2 groups), family history of breast cancer (2 groups) and cigarette smoking (3 groups) were incorporated into the model as possible confounding variables, although, in the event, they had little effect on the estimation of relative risks (Vessey *et al.*, 1979). Separate analyses were made within 4 age groups (16–35, 36–40, 41–45, 46–50) and 3 parity groups (0, 1–2, ⩾3 children) as well as for all the data combined. Table VII summarises the results obtained overall and according to age. In the three younger age groups, there are two statistically significant values of $\chi^2$ for heterogeneity but no patterns of risk emerge and it seems reasonable to ascribe these apparent

**Table VII** Risk of breast cancer in relation to different measures of exposure to oral contraceptives by age. In each analysis, risk among women never using oral contraceptives is taken as unity (95% confidence limits are given in parentheses)

| | *Age group (years)* | | | | |
|---|---|---|---|---|---|
| | *16–35* | *36–40* | *41–45* | *46–50* | *All ages* |
| *No. of pairs* | *210* | *257* | *388* | *321* | *1176* |
| Use at any time: | | | | | |
| No | 1.00 | 1.00 | 1.00 | 1.00 | 1.00 |
| Yes | 0.94 (0.57–1.53) | 0.86 (0.56–1.31) | 0.72 (0.51–1.02) | 1.50 (1.04–2.16) | 0.98 (0.81–1.18) |
| $\chi_1^2$ heterogeneity | 0.07 | 0.51 | 3.41 | 4.77* | 0.05 |
| Interval since last used (months) | | | | | |
| Never | 1.00 | 1.00 | 1.00 | 1.00 | 1.00 |
| -12 | 0.93 | 0.85 | 0.78 | 1.20 | 0.99 (0.76–1.30) |
| 13–48 | 0.92 | 0.74 | 0.82 | 1.27 | 0.95 (0.70–1.31) |
| 49–96 | 1.23 | 1.42 | 0.99 | 1.86 | 1.34 (0.98–1.83) |
| 97+ | 0.54 | 0.40 | 0.38 | 1.74 | 0.67 (0.48–0.94) |
| $\chi_4^2$ heterogeneity | 2.20 | 7.80 | 11.08* | 6.09 | 10.50* |
| Interval since first used (months) | | | | | |
| Never | 1.00 | 1.00 | 1.00 | 1.00 | 1.00 |
| -48 | 0.58 | 0.98 | 0.62 | 1.31 | 0.83 (0.59–1.17) |
| 49–96 | 1.24 | 1.14 | 0.84 | 1.82 | 1.20 (0.91–1.59) |
| 97–144 | 1.05 | 0.79 | 1.15 | 1.80 | 1.17 (0.87–1.55) |
| 145+ | 1.32 | 0.55 | 0.43 | 1.23 | 0.73 (0.54–0.99) |
| $\chi_4^2$ heterogeneity | 5.59 | 3.94 | 12.20* | 6.24 | 10.29* |
| $\chi_1^2$ linear trend | 0.55 | 2.03 | 4.85* | 3.25 | 0.44 |
| Total duration of use (months) | | | | | |
| Never | 1.00 | 1.00 | 1.00 | 1.00 | 1.00 |
| -12 | 0.79 | 0.70 | 0.60 | 1.72 | 0.88 (0.69–1.13) |
| 13–48 | 0.76 | 1.29 | 0.76 | 1.71 | 1.01 (0.76–1.33) |
| 49–96 | 1.62 | 0.87 | 0.71 | 1.28 | 1.16 (0.84–1.59) |
| 97+ | 1.01 | 0.71 | 1.01 | 1.07 | 0.99 (0.67–1.45) |
| $\chi_4^2$ heterogeneity | 6.73 | 3.67 | 5.52 | 6.11 | 2.43 |
| $\chi_1^2$ linear trend | 1.08 | 0.18 | 0.48 | 1.71 | 0.22 |

*$P<0.05$.

Risks adjusted for effects of social class, age at menarche, age at first term birth, menopausal state, smoking habits, history of breast biopsy and family history of breast cancer.

associations to chance. The increased relative risk associated with oral contraceptive use in those aged 46–50 y (1.50:1) was also statistically significant, although it should be noted that the biggest deficiency of use in breast cancer patients occurred in those just 5 years younger. In our last report (Vessey *et al.*, 1979), the relative risk for the 46–50 y age group (based on 115 case-control pairs) was 2.40:1 while the relative risk based on the 206 case-control pairs interviewed since then is 1.39:1—i.e. much smaller, but still elevated. There is, however, little evidence of any pattern in the relative risks in the 46–50 y age group in the remainder of Table VII—in particular, there is no evidence that either very long durations of oral contraceptive use or use in the very distant past is associated with an increased risk. Despite this, we decided to take a close look at oral contraceptive use in women aged 46–50 y (and, for comparative purposes, in those aged 41–45 y) in relation to menopausal state. Some simple results are given in Table VIII from which it can be seen that the slight excess of oral contraceptive use among women with breast cancer in the older age group is apparent in all three menopause subcategories. More sophisticated analysis of the data using the method of Breslow *et al.* (1978) confirmed that menopausal state was not an important "modifier" of the effect of oral contraceptive use in those aged 46–50 y.

In a second series of analyses, we examined the effects of different types of oral contraceptives. Again, separate analyses were made within age and parity subgroups as well as overall. We were unable to discern any pattern in the large array of relative risks calculated. In particular, the two associations which were statistically significant in our last analysis (an increased risk associated with preparations containing 75–80 μg oestrogen in women aged 36–40 y and a decreased risk associated with preparations of unknown type in women aged 41–45 y) were not apparent in the data collected during the last 3 years of the study.

Finally, we searched for evidence of any interactions between oral contraceptive use and late age at first term birth, a history of breast biopsy, and a family history of breast cancer. Table IX gives the basic data (subdivided by total duration of use) which provide little indication of any effect; this was confirmed by multivariate analysis. The numbers of subjects included in most of the analyses were, however, small despite the large size of the study overall.

Use of oral contraceptives before the first term birth has been the subject of a separate publication (Vessey *et al.*, 1982). No evidence of any deleterious effect was found.

### *Use of hormone replacement therapy*

During the last 3 years of the study, questions were asked about the use of hormone replacement therapy as well as about oral contraceptive use. As expected, use of such therapy was infrequent. Among 555 women with breast cancer, 34 (6.1%) reported use of replacement therapy of whom 8 (1.4%) reported more than one year's use. Among the controls, the corresponding figures were 37 (6.7%) and 11 (2.0%).

### *Diagnostic bias*

In our last general report (Vessey *et al.*, 1979) we presented data for users and non-users of oral contraceptives about delay in seeking treatment for breast cancer, about the identity of the individual who first discovered the tumour and about the prevalence of regular breast self examination. We

**Table VIII** Oral contraceptive use by age and menopausal state

| *Age* | *Menopausal state* | *No. cases* | *% ever using pill* | *% with recent pill use** | *% using pill >48 mo.* | *No. controls* | *% ever using pill* | *% with recent pill use** | *% using pill >48 mo.* |
|---|---|---|---|---|---|---|---|---|---|
| 41–45 | Pre | 355 | 36.3 | 11.8 | 14.9 | 293 | 42.3 | 13.3 | 15.7 |
| | Natural | 7 | 0 | 0 | 0 | 18 | 16.7 | 0 | 5.6 |
| | Artificial | 26 | 50.0 | 3.8 | 15.4 | 77 | 45.5 | 1.3 | 7.8 |
| | Total | 388 | 36.6 | 11.1 | 14.7 | 388 | 41.8 | 10.3 | 13.7 |
| 46–50 | Pre | 220 | 36.8 | 10.9 | 15.0 | 167 | 32.3 | 9.6 | 12.0 |
| | Natural | 49 | 28.6 | 0 | 8.2 | 69 | 21.7 | 7.2 | 5.8 |
| | Artificial | 52 | 40.4 | 3.8 | 3.8 | 85 | 30.6 | 2.4 | 7.1 |
| | Total | 321 | 36.1 | 8.1 | 12.1 | 321 | 29.6 | 7.2 | 9.3 |

*"Recent" pill use—use within the 12 months prior to diagnosis.

**Table IX** Total duration of oral contraceptive use in various subgroups of patients with breast cancer and controls

| *Duration of oral contraceptive use (mo.)* | *Number* (%) *with cancer* | *Number* (%) *of controls* |
|---|---|---|
| Parous patients aged 26 or more at 1st term birth | | |
| Never | 284 (64.0) | 181 (59.0) |
| ≤48 | 107 (24.1) | 93 (30.3) |
| 49+ | 53 (11.9) | 33 (10.7) |
| Total | 444 (100.0) | 307 (100.0) |
| Patients with history of breast biopsy | | |
| Never | 67 (65.0) | 41 (56.9) |
| ≤48 | 29 (28.2) | 25 (34.7) |
| 49+ | 7 (6.8) | 6 (8.3) |
| Total | 103 (100.0) | 72 (100.0) |
| Patients with family history of breast cancer | | |
| Never | 62 (59.0) | 32 (52.5) |
| ≤48 | 26 (24.8) | 21 (34.4) |
| 49+ | 17 (16.2) | 8 (13.1) |
| Total | 105 (100.0) | 61 (100.0) |

came to the conclusion that there was little evidence that anxiety about the possible relationship between pill use and breast cancer was leading to diagnostic bias. The additional data available in the present analysis (not shown) have not altered this conclusion.

*Clinical stage of breast tumours*

Table X gives the clinical stage classification of 572 of the 582 patients treated for breast cancer at the London hospitals up to the end of August 1977; no stage could be assigned to the remaining 10 because of inadequate clinical information in the records. As before, women who had never used oral contraceptives had appreciably more advanced tumours than those who had been using oral contraceptives during the year before detection of the lump ("recent" users), while past users were in an intermediate position.

*Mortality of patients with breast tumours*

Of the 572 women with breast cancer included in Table X, 562 were followed until the end of December 1979. There were 182 deaths. All 10 patients who were not followed had emigrated; no attempt was made to trace them after their departure.

Table XI gives an analysis of mortality, using the log rank method (Peto *et al.*, 1977), in relation to oral contraceptive use at the time the tumour was first detected. As in our last analysis, recent users of the pill had a lower mortality than past users, who in turn had a lower mortality than those who had never used the pill. These differences were, however, small and not statistically significant; they almost entirely disappeared after allowance had been made for the effect of clinical stage on survival.

### Discussion

Since the appearance of our last detailed report (Vessey *et al.*, 1979), the results of a considerable number of additional epidemiological studies of the possible association between oral contraceptive use and breast cancer have been published (Paffenbarger *et al.*, 1979; Ravnihar *et al.*, 1979; Jick *et al.*, 1980; Matthews *et al.*, 1981; Pike *et al.*, 1981; Royal College of General Practitioners, 1981; Vessey *et al.*, 1981; Trapido, 1981; Harris *et al.*,

**Table X** Stage classification of 572 patients with breast cancer (TNM system) treated at the London hospitals before the end of August, 1977. Figures are numbers of patients (percentages in parentheses)

| *Clinical stage* | *Use of oral contraceptives** *Never used* | *Used only in past* | *Used recently* |
|---|---|---|---|
| I ($T_{1-2}$, $N_0$, $M_0$) | 196 (55.4) | 83 (64.3) | 66 (74.2) |
| II ($T_{1-2}$, $N_1$, $M_0$) | 74 (20.8) | 23 (17.8) | 11 (12.4) |
| III–IV (other TNM categories) | 84 (23.8) | 23 (18.0) | 12 (13.4) |
| Total | 354 (100.0) | 129 (100.0) | 89 (100.0) |

*"Used recently" indicates use during year before detection of lump. "Used only in past" indicates use only before that time.

$\chi^2_{(4)} = 11.75$; $P = 0.02$.

**Table XI** Mortality among 572 patients with breast cancer treated at the London hospitals before the end of August, 1977. Closure date for analysis 31st December, 1979. Analysis by log rank method (Peto *et al.*, 1977)

| | *Use of oral contraceptives:* | | | |
|---|---|---|---|---|
| *Number of deaths:* | *Never used* | *Used only in past* | *Used recently* | $\chi^2_{(1)}$ *linear trend* |
| Observed | 120 | 37 | 25 | — |
| Expected: | | | | |
| Unadjusted for stage | 112.5 | 39.0 | 30.5 | 1.60 (NS) |
| Adjusted for stage | 118.1 | 36.8 | 27.1 | 0.17 (NS) |

1982). The results of these studies have mostly been reassuring, but Jick *et al.* (1981) suggested that recent (within a year) oral contraceptive use might be hazardous in premenopausal women aged 46–55 y, while Pike *et al.* (1981) and Harris *et al.* (1982) found an increased risk to be associated with oral contraceptive use before first full term pregnancy. In the study by Pike *et al.* (1981), which dealt only with women with breast cancer aged up to 32 y, this harmful effect was apparent only after 4 years pill use. Our data (both those appearing in the present report and those appearing in Vessey *et al.* (1982)) offer no support to these positive results and, on balance, give no cause for concern. A few statistically significant differences in oral contraceptive use were found between the breast cancer and control groups, but the data were subdivided in so many ways that some "significant" differences would have been expected to occur through the play of chance alone. Certainly no patterns of risk have emerged which seem to us to point towards a causal association between pill use and the occurrence of breast cancer. It must be stressed, however, that the data are still sparse in some important sub categories—for example, only small numbers of both cases and controls in our study had prolonged oral contraceptive use before their first term pregnancy. For this reason, it is important that data on the possible relationship between oral contraceptive use and breast cancer should continue to be collected.

A large, carefully designed, and well-conducted case-control study of oral contraceptive use and breast cancer, funded by the National Institutes of Health, is at present being conducted by the Family Planning Evaluation Division of the Centers for Disease Control in Atlanta. In contrast with most other case-control studies of this question, this investigation is using community controls rather than hospital controls. Although no formal paper describing the preliminary results of the study has yet been published, a number of reports concerning the first 689 cases and 1072 controls have appeared indicating that no evidence has been found that breast cancer risk is influenced by pill use (Anonymous, 1982).

In our last full analysis (Vessey *et al.*, 1979), we noted that a smaller proportion of women with breast cancer than of matched controls reported never having used any method of birth control at all. The addition of data for a further 555 pairs of patients has left this association unchanged. As a possible explanation, we suggested in 1979 that infertile women, who have less need for contraception, might be less likely to develop breast cancer than fertile women. Our examination of the characteristics of the 120 breast cancer patients and 190 controls who did not admit to the use of birth control, however, makes this explanation unlikely—only 25% of the former and 19% of the latter had never had any children. We are therefore at something of a loss to explain the difference, although it might, perhaps, mean that women with breast cancer, because of the nature of their illness, are more likely to admit to the use of contraception than controls. Certainly it could be argued that the risks of breast cancer associated with oral contraception should be estimated only from data relating to patients and controls admitting to the use of some birth control method. We have chosen not to adopt this approach in the present paper but it may be noted that, on this basis, the adjusted relative risk of breast cancer among those with any use of oral contraceptives is only 0.80 (as opposed to 0.98—see Table VII).

The negative association between cigarette smoking and breast cancer was noted in our last full report and has now been consolidated by the collection of additional data. We have been unable to explain the association in terms of any known confounding variable. It is, of course, established that, on average, cigarette smokers have an earlier age of natural menopause than non-smokers (Jick *et al.*, 1977) and, as a consequence, post-menopausal smokers would be expected to be at slightly lower risk of breast cancer than non-smokers. The association that we have observed cannot, however, be explained in terms of this mechanism. It is also possible that smoking might offer some additional protection against breast cancer (MacMahon *et al.*, 1982; Baron J., personal communication), but we suspect that the unrepresentative nature of smoking habits amongst our hospital controls—in some of whom cigarette smoking is likely to have contributed to their ill-health—is a likely explanation for our results.

Finally, the small amount of additional information in the present report on clinical stage of breast tumours at diagnosis supports our earlier observation of a negative association between stage and oral contraceptive use. Once again, we found no evidence that this association can be attributed to "surveillance bias" among oral contraceptive users and the possibility of a beneficial effect of contraceptive steroids on tumour growth and spread must be considered.

We thank the medical staff at the participating hospitals for allowing us to study patients under their care and Miss K. Jones, Mrs M. Simmonds, Mrs. J. Young and Mrs. K. Rodriguez for conducting the interviews and following up the patients. We are also grateful to the Medical Research Council and the Imperial Cancer Research Fund for financial support.

### References

ANONYMOUS, (1982). Pill does not increase risk of breast cancer even after years of use. *Family Planning Perspectives*, **14**, 216.

BRESLOW, N.E., DAY, N.E., HALVORSEN, K.T., PRENTICE, R.L. & SABAI, C. (1978). Estimation of multiple relative risk functions in matched case-control studies. *Am. J. Epidemiol.*, **108**, 299.

HARRIS, N.V., WEISS, N.S., FRANCIS, A.M. & POLLISSAR, L. (1982). Breast cancer in relation to patterns of oral contraceptive use. *Am. J. Epidemiol.*, **116**, 643.

INTERNATIONAL UNION AGAINST CANCER (1968). TNM classification of malignant tumours. Geneva. UICC.

JICK, H., PORTER, J. & MORRISON, A.S. (1977). Relation between smoking and age of natural menopause. *Lancet*, **i**, 1354.

JICK, H., WALKER, A.M., WATKINS, R.N., D'EWART, D.C., HUNTER, J.R. *et al.* (1980). Oral contraceptives and breast cancer. *Am. J. Epidemiol.*, **112**, 577.

KALACHE, A. & VESSEY, M.P. (1982). Risk factors for breast cancer. *Clinics in Oncology*, **1**, 661.

MACMAHON, B., TRICHOPOULOS, D., COLE, P. & BROWN, J. (1982). Cigarette smoking and urinary estrogens. *New Engl. J. Med.*, **307**, 1062.

MATTHEWS, D.N., MILLIS, R.R. & HAYWARD, J.L. (1981). Breast cancer in women who have taken contraceptive steroids. *Br. Med. J.*, **282**, 774.

PAFFENBARGER, R.S., KAMPERT, J.B. & CHANG, H. (1979). Oral contraceptives and breast cancer risk. *Les Éditions de L'Institut National de la Santé et de la Recherche Médicale*, **83**, 185.

PETO, R., PIKE, M.C., ARMITAGE, P., BRESLOW, N.E., COX, D.R. *et al.* (1977). Design and analysis of randomised clinical trials requiring prolonged observation of each patient. ii Analysis & examples. *Br. J. Cancer*, **35**, 1.

PIKE, M.C., HENDERSON, B.E., CASAGRANDE, J.T., ROSARIO, I. & GRAY, G.E. (1981). Oral contraceptive use and early abortion as risk factors for breast cancer in young women. *Br. J. Cancer*, **43**, 72.

RAVNIHAR, B., SEIGEL, D.G. & LINDTNER, J. (1979). An epidemiologic study of breast cancer and benign breast neoplasias in relation to the oral contraceptive and estrogen use. *Eur. J. Cancer*. **15**, 295.

ROYAL COLLEGE OF GENERAL PRACTITIONERS (1981). Breast cancer and oral contraceptives: findings in the Royal College of General Practitioners Study. *Br. Med. J.*, **282**, 2088.

TRAPIDO, E.J. (1981). A prospective cohort study of oral contraceptives and breast cancer. *J. Natl Cancer Inst.*, **67**, 1011.

VESSEY, M.P., DOLL, R. & SUTTON, P.M. (1972). Oral contraceptives and breast neoplasia: a retrospective study. *Br. Med. J.*, **iii**, 719.

VESSEY, M.P., DOLL, R. & JONES, K. (1975). Oral contraceptives and breast cancer: progress report of an epidemiological study. *Lancet*, **i**, 941.

VESSEY, M.P., DOLL, R., JONES, K., MCPHERSON, K. & YEATES, D. (1979). An epidemiological study of oral contraceptives and breast cancer. *Br. Med. J.*, **i**, 1755.

VESSEY, M.P., MCPHERSON, K. & DOLL, R. (1981). Breast cancer and oral contraceptives: findings in Oxford-Family Planning Association contraceptive study. *Br. Med. J.*, **282**, 2093.

VESSEY, M.P., MCPHERSON, K., YEATES, D. & DOLL, R. (1982). Oral contraceptive use and abortion before first term pregnancy in relation to breast cancer risk. *Br. J. Cancer*, **45**, 327.